# Supplementary material for: Human-Induced Pluripotent Stem Cell (iPSC)-Derived GABAergic Neuron Differentiation in Bipolar Disorder
Source: Cells. 2024 Jul 15;13(14):1194. doi: 10.3390/cells13141194 (PMC11275104; doi:10.3390/cells13141194)
Supplement: Supplementary file 1 [file cells-13-01194-s001.zip › cells-3049147-supplementary.pdf]

5. Supplemental data

SUPPLEMENTAL TABLE S1 PRIMER SEQUENCES	
RPL27 F -GTGGATATCCCCTTGGACAA	RPL27 R -TCAAACTTGACCTTGGCCTC
GAD67 F-TCAAGTAAAGATGGTGATGGGATA	GAD67 R -GCCATGATGCTGTACATGTTG
RBFox3B (NeuN) F- GGGGAAGGCAGTGACTAGGT	RBFox3B (NeuN) R - CGCAGCCCGAAATGTATTAT
CACNA1C (Cav1.2α) F-TGGTCAATGAGAATACGAGG	CACNA1C (Cav1.2α) R - CCATAGTTGGAACCTTGGTG
SLC12A2 (KCC1) F - TAAAGGAGTCGTGAAGTTTGGC	SLC12A2 (NKCC1) R - CTTGACCCACAATCCATGACA
SLC12A4 (NKCC1) F - CCTCCCGTGTTTCCGGTATG	SLC12A4 (KCC1) R - CAGGAGTCGGTCGTAAGGTTG
SLC12A5 (KCC2) F - CTGGCCAACTACACCAACCT	SLC12A5 (KCC2) R - GAGGATGACGCCAAAGATGT
SST F-GCTGCTGTCTGAACCCAAC	SST R -CGTTCTCGGGGTGCCATAG
S100β F - TAATCTCACTCATGTTCAAAGAACTCGTG	S100β R -ATGGAGACGGCGAATGTGACT
vGlut1 F –GAAGCTGCACCGCCTTCT	vGlut1 R –CAGACCACTCATGATGGCGA

Supplemental Table S2.

**Comparison**	**P-value**	**Genes**
Crispr vs Control	0.01	183
Crispr vs Control	0.01	68
Crispr vs Control	0.01	115
BP vs C	0.01	293
BP vs C	0.01	69
BP vs C	0.01	224
Crispr vs BP	0.01	120
Crispr vs BP	0.01	66
Crispr vs BP	0.01	54

Supplemental Table S3.

WEEK	COMPARISON	NKCC1		KCC2		RATIO	
		F	t	F	t	F	t
1	C v BP	-	-	-	-	0.045	-
	C v BP-C	-	-	0.004	-	-	-
	BP v BP-C	-	-	0.006	-	-	-
2	C v BP	-	-	-	-	-	-
	C v BP-C			0.043			
	BP v BP-C	-	-	0.017			
3	C v BP	-	-	0.033		0.009	0.047
	C v BP-C	-	-	-	-	-	-
	BP v BP-C	-	-	-	-	0.011	0.055
4	C v BP	0.049	-	-	-	-	-
	C v BP-C	0.015	0.016	-	-	-	0.055
	BP v BP-C	0.015					
5	C v BP	-	-	0.061	-	-	-
	C v Bp-C	-	-	0.004	-	-	-
	BP v BP-C	-	-	-	-	-	-
6	C v BP	-	0.032	-	0.069	0.042	0.065
	C v BP-C	-	-	-	0.044	-	-
	BP-BP-C	0.005	-	-	-	-	-

Supplemental Table S3. Significance testing of means and variances of expression of NKCC1, KCC2 or of the ratio of NKCC1/NKCC2 at weekly intervals through the 6-week differentiation of GABA differentiation qRT-PCR analysis. P values were calculated using ANOVA (F test), and means tests done with paired t-tests. Data were collected over week 1 through

Supplemental Text

Main categories of **differentially expressed genes (DEG)** between bipolar and control neurons include regulators of the extracellular milieu: Bone morphogenetic protein 1 (BMP1), Mannose phosphate isomerase (MPI), Laminin alpha4 (LAMA4), Neuron derived neurotrophic factor (NDNF), and Sulfatase 1 (SULF1). BMP1, a metalloprotease that regulates the extracellular matrix, plays developmental and homeostatic roles in various tissues, including the nervous system. Sonic hedgehog (Shh) and BMPs form a ventral to dorsal gradient morphogens in the developing CNS. As in our differentiation protocol Shh contributes to GABAergic neuronal differentiation in the early stages of development with BMPs contributing as cells mature [36] One of BMP1's substrates is THBS1, thromobospondin-1, which we have identified as a protein of interest in bipolar astrocyte derived exosomes. A nonsense mutation in BMP1 was also identified in offspring of a bipolar disorder patient [37]. LAMA4 is an extracellular matrix protein that plays roles in numerous biological processes including neurite outgrowth [38]. Mutations of LAMA4 have been identified in familial bipolar disorder via exome sequencing [39 LAMA4 was also identified as a hub gene in an analysis of differentially expressed genes in bipolar disorder patients who are lithium responders [40]. NDNF is a secreted protein that promotes neuronal migration, and neurite outgrowth [41]. Similarly SULF1, an extracellular heparan sulfate endosulfatase, contributes to synapse formation and function [42].

Differentially expressed genes involved in the regulation of synapses include SPRX2, Leucine rich repeat kinase 2 (LRKK2), and SSTR3. SPRX2 protects developing synapses from complement mediated elimination [43] while mice lacking SPRX2 demonstrate neurobehavioral / synaptic abnormalities [44]. LRRK2 is a serine / threonine kinase that modulates neural plasticity [45] interacts with neuronal vesicles [46], and may play a role in Shh signaling in the brain via regulation of ciliogenesis [47]. LRKK2 is mutated in Parkinson's patients and a subset of those patients have also been diagnosed with bipolar disorder [48]. Like LRRK2, the somatostatin receptor

SSTR3 contributes to neuronal cilia which have been implicated in neurological disorders [49,50]. SST is robustly expressed in our GABAergic neurons (Figure 2), and this subtype of GABAergic neurons have been shown to express SSTR3 in other contexts [51]. Finally, the FOXP1 gene linked to neurodevelopmental disorders encodes risk factors that regulate the generation of GABA neurons and E/I balance in the cortex; a common mechanism for neurodegenerative disorders is reduction in the number of GABAergic interneurons.

Differentially expressed genes of interest that are either ion channels or cotransporters include the (glutamate ionotrophic receptor NMDA type subunit 2A (GRIN2A), dipeptidyl peptidase like 10 (DPP10), and solute carrier family 12 member 8 (SLC12A8). GRIN2A is a glutamate-gated ion channel protein that has been shown to be downregulated in bipolar disorder and is upregulated following lithium treatment [52]. DPP10 binds voltage gated potassium channels impacting their function and expression [53]. Mutations and copy number variations in DPP10 have been described in both bipolar disorder and schizophrenia [54,55]. Significant numbers of SNPs in SLC12A8, a cation / chloride cotransporter, have been identified in schizophrenia patients [56]. A paralog of this gene SLC12A2 (NKCC1) demonstrates a similar expression pattern in both RNA-sequencing and qRT-PCR (Figures 2, 3, 6).

There were several differentially expressed genes of interest that do not fit into these categories including: Beta-secretase (BACE), FER3 like bHLH transcription factor (FERD3L), and annexinA4 (ANXA4). BACE2, an aspartic protease that cleaves amyloid precursor proteins, has been implicated in both Alzheimer's and Down Syndrome [57,58]. FERD3L, (Fer3like bHLH transcription factor) previously known as Nato3 and N-Twist, is a basic helix loop helix transcription factor that contributes to dopaminergic neuron specification and neurogenesis [59,60]. Polymorphisms in this gene have been associated with psychiatric disorders ([61]. Annexin A4, (ANXA4), a calcium-dependent phospholipid binding protein has previously been linked to bipolar disorder. ANXA4 was identified in a GWAS study of bipolar disorder patients [62] and was reported to be differentially expressed in cells derived from monozygotic twins discordant for bipolar disorder [63]. Other Annexins, ANXA1 and ANXA11, demonstrate similar expression patterns in our RNA-sequencing data set as well (Figure 3).

There are several additional genes that are upregulated in bipolar GABAergic neurons as compared to controls including, LRCC37A2, WRN helicase 37A2, (WRNIP1), and SOX4. LRCC37A2, a membrane protein associated with epilepsy and schizophrenia [64,65]. Differential expression of LRCC37A2 in GABAergic neurons via single nuclei RNA seq in a cohort of 75 nonaged adults has been reported [66] but this has not been demonstrated in

the context of bipolar disorder. WRNIP1 is a helicase interacting protein that accumulates at sites of DNA damage. WRNIP1 and WRN that it interacts with it are associated with Werner syndrome, a disease of premature aging [67]. A SNP in WRN, 1367R, was reported to be associated with bipolar disorder [68]. SOX4 (SRY-box) transcription factor 4 lrcc37a2 has demonstrated roles in central nervous system development [69]. Importantly, overexpression of SOX4 results in premature induction of neuronal marker genes and promotes neurogenesis [70,71].

Supplemental References

36. Yung, S.Y.; Gokhan, S.; Jurcsak, J.; Molero, A.E.; Abrajano, J.J.; Mehler, M.F. Differential modulation of BMP signaling promotes the elaboration of cerebral cortical GABAergic neurons or oligodendrocytes from a common sonic hedgehog-responsive ventral forebrain progenitor species. *Proc. Natl. Acad. Sci. USA* **2002**, *99*, 16273–16278. https://doi.org/10.1073/pnas.232586699.
37. Toma, C.; Shaw, A.D.; Allcock RJ, N.; Heath, A.; Pierce, K.D.; Mitchell, P.B.; Schofield, P.R.; Fullerton, J.M. An examination of multiple classes of rare variants in extended families with bipolar disorder. *Transl. Psychiatry* **2018**, *8*, 65. https://doi.org/10.1038/s41398-018-0113-y.
38. Ichikawa, N.; Kasai, S.; Suzuki, N.; Nishi, N.; Oishi, S.; Fujii, N.; Kadoya, Y.; Hatori, K.; Mizuno, Y.; Nomizu, M.; et al. Identification of neurite outgrowth active sites on the laminin alpha4 chain G domain. *Biochemistry* **2005**, *44*, 5755–5762. https://doi.org/10.1021/bi0476228.
39. Goes, F.S.; Pirooznia, M.; Parla, J.S.; Kramer, M.; Ghiban, E.; Mavruk, S.; Chen, Y.C.; Monson, E.T.; Willour, V.L.; Karchin, R.; et al. Exome Sequencing of Familial Bipolar Disorder. *JAMA Psychiatry* **2016**, *73*, 590–597. https://doi.org/10.1001/jamapsychiatry.2016.0251.
40. Niemsiri, V.; Rosenthal, S.B.; Nievergelt, C.M.; Maihofer, A.X.; Marchetto, M.C.; Santos, R.; Shekhtman, T.; Alliey-Rodriguez, N.; Anand, A.; Balaraman, Y.; et al. Focal adhesion is associated with lithium response in bipolar disorder: Evidence from a network-based multi-omics analysis. *Mol. Psychiatry* **2023**, *29*, 6–19. https://doi.org/10.1038/s41380-022-01909-9.
41. Kuang, X.L.; Zhao, X.M.; Xu, H.F.; Shi, Y.Y.; Deng, J.B.; Sun, G.T. Spatio-temporal expression of a novel neuron-derived neurotrophic factor (NDNF) in mouse brains during development. *BMC Neurosci.* **2010**, *11*, 137. https://doi.org/10.1186/1471-2202-11-137.
42. Kamimura, K.; Maeda, N. Glypicans and Heparan Sulfate in Synaptic Development, Neural Plasticity, and Neurological Disorders. *Front. Neural Circuits* **2021**, *15*, 595596. https://doi.org/10.3389/fncir.2021.595596.
43. Cong, Q.; Soteros, B.M.; Wollet, M.; Kim, J.H.; Sia, G.M. The endogenous neuronal complement inhibitor SRPX2 protects against complement-mediated synapse elimination during development. *Nat. Neurosci.* **2020**, *23*, 1067–1078. https://doi.org/10.1038/s41593-020-0672-0.
44. Soteros, B.M.; Cong, Q.; Palmer, C.R.; Sia, G.M. Sociability and synapse subtype-specific defects in mice lacking SRPX2, a language-associated gene. *PLoS ONE* **2018**, *13*, e0199399. https://doi.org/10.1371/journal.pone.0199399.
45. Zach, S.; Felk, S.; Gillardon, F.Signal transduction protein array analysis links LRRK2 to Ste20 kinases and PKC zeta that modulate neuronal plasticity. *PLoS ONE* **2010**, *5*, e13191. https://doi.org/10.1371/journal.pone.0013191.
46. Piccoli, G.; Onofri, F.; Cirnaru, M.D.; Kaiser, C.J.; Jagtap, P.; Kastenmüller, A.; Pischedda, F.; Marte, A.; von Zweydorf, F.; Vogt, A.; et al. Leucine-rich repeat kinase 2 binds to neuronal vesicles through protein interactions mediated by its C-terminal WD40 domain. *Mol. Cell. Biol.* **2014**, *34*, 2147–2161. https://doi.org/10.1128/MCB.00914-13.
47. Steger, M.; Diez, F.; Dhekne, H.S.; Lis, P.; Nirujogi, R.S.; Karayel, O.; Tonelli, F.; Martinez, T.N.; Lorentzen, E.; Pfeffer, S.R.; et al. Systematic proteomic analysis of LRRK2-mediated Rab GTPase phosphorylation establishes a connection to ciliogenesis. *eLife* **2017**, *6*, e31012. https://doi.org/10.7554/eLife.31012.
48. Shanker, V.; Groves, M.; Heiman, G.; Palmese, C.; Saunders-Pullman, R.; Ozelius, L.; Raymond, D.; Bressman, S. Mood and cognition in leucine-rich repeat kinase 2 G2019S Parkinson's disease. *Mov. Disord. Off. J. Mov. Disord. Soc.* **2011**, *26*, 1875–1880. https://doi.org/10.1002/mds.23746.
49. Alhassen, W.; Chen, S.; Vawter, M.; Robbins, B.K.; Nguyen, H.; Myint, T.N.; Saito, Y.; Schulmann, A.; Nauli, S.M.; Civelli, O.; et al. Patterns of cilia gene dysregulations in major psychiatric disorders. *Progress. Neuro-Psychopharmacol. Biol. Psychiatry* **2021**, *109*, 110255. https://doi.org/10.1016/j.pnpbp.2021.110255.

50. Song, Y.H.; Yoon, J.; Lee, S.H. The role of neuropeptide somatostatin in the brain and its application in treating neurological disorders. *Exp. Mol. Med.* **2021**, *53*, 328–338. https://doi.org/10.1038/s12276-021-00580-4.
51. Lukomska, A.; Dobrzanski, G.; Liguz-Lecznar, M.; Kossut, M. Somatostatin receptors (SSTR1-5) on inhibitory interneurons in the barrel cortex. *Brain Struct. Funct.* **2020**, *225*, 387–401. https://doi.org/10.1007/s00429-019-02011-7.
52. Akkouh, I.A.; Skrede, S.; Holmgren, A.; Ersland, K.M.; Hansson, L.; Bahrami, S.; Andreassen, O.A.; Steen, V.M.; Djurovic, S.; Hughes, T. Exploring lithium's transcriptional mechanisms of action in bipolar disorder: A multi-step study. *Neuropsychopharmacology* **2020**, *45*, 947–955. https://doi.org/10.1038/s41386-019-0556-8.
53. Jerng, H.H.; Lauver, A.D.; Pfaffinger, P.J. DPP10 splice variants are localized in distinct neuronal populations and act to differentially regulate the inactivation properties of Kv4-based ion channels. *Mol. Cell. Neurosci.* **2007**, *35*, 604–624. https://doi.org/10.1016/j.mcn.2007.03.008.
54. Gonzalez, S.; Villa, E.; Rodriguez, M.; Ramirez, M.; Zavala, J.; Armas, R.; Dassori, A.; Contreras, J.; Raventós, H.; Flores, D.; et al. Fine-mapping scan of bipolar disorder susceptibility loci in Latino pedigrees. *Am. J. Med. Genetics. Part B Neuropsychiatr. Genet. Off. Publ. Int. Soc. Psychiatr. Genet.* **2019**, *180*, 213–222. https://doi.org/10.1002/ajmg.b.32715.
55. Lachman, H.M. Copy variations in schizophrenia and bipolar disorder. *Cytogenet. Genome Res.* **2008**, *123*, 27–35. https://doi.org/10.1159/000184689.
56. Zhuo, C.; Tian, H.; Chen, J.; Li, Q.; Yang, L.; Zhang, Q.; Chen, G.; Cheng, L.; Zhou, C.; Song, X. Associations of cognitive impairment in patients with schizophrenia with genetic features and with schizophrenia-related structural and functional brain changes. *Front. Genet.* **2022**, *13*, 880027. https://doi.org/10.3389/fgene.2022.880027.
57. Wang, Z.; Xu, Q.; Cai, F.; Liu, X.; Wu, Y.; Song, W. BACE2, a conditional β-secretase, contributes to Alzheimer's disease pathogenesis. *JCI Insight* **2019**, *4*, e123431. https://doi.org/10.1172/jci.insight.123431.
58. Alić, I.; Goh, P.A.; Murray, A.; Portelius, E.; Gkanatsiou, E.; Gough, G.; Mok, K.Y.; Koschut, D.; Brunmeir, R.; Yeap, Y.J.; et al. Patient-specific Alzheimer-like pathology in trisomy 21 cerebral organoids reveals BACE2 as a gene dose-sensitive AD suppressor in human brain. *Mol. Psychiatry* **2021**, *26*, 5766–5788. https://doi.org/10.1038/s41380-020-0806-5.
59. Verzi, M.P.; Anderson, J.P.; Dodou, E.; Kelly, K.K.; Greene, S.B.; North, B.J.; Cripps, R.M.; Black, B.L. N-Twist, an Evolutionarily Conserved bHLH Protein Expressed in the Developing CNS, Functions as a Transcriptional Inhibitor. *Dev. Biol.* **2002**, *249*, 174–190. https://doi.org/10.1006/DBIO.2002.0753.
60. Ono, Y.; Nakatani, T.; Minaki, Y.; Kumai, M. The basic helix-loop-helix transcription factor Nato3 controls neurogenic activity in mesencephalic floor plate cells. *Development* **2010**, *137*, 1897–1906. https://doi.org/10.1242/dev.042572.
61. Jukic, M.M.; Carrillo-Roa, T.; Bar, M.; Becker, G.; Jovanovic, V.M.; Zega, K.; Binder, E.B.; Brodski, C. Abnormal development of monoaminergic neurons is implicated in mood fluctuations and bipolar disorder. *Neuropsychopharmacol. Off. Publ. Am. Coll. Neuropsychopharmacol.* **2015**, *40*, 839–848. https://doi.org/10.1038/npp.2014.244.
62. Smith, E.N.; Bloss, C.S.; Badner, J.A.; Barrett, T.; Belmonte, P.L.; Berrettini, W.; Byerley, W.; Coryell, W.; Craig, D.; Edenberg, H.J.; et al. Genome-wide association study of bipolar disorder in European American and African American individuals. *Mol. Psychiatry* **2009**, *14*, 755–763. https://doi.org/10.1038/mp.2009.43.
63. Kazuno, A.A.; Ohtawa, K.; Otsuki, K.; Usui, M.; Sugawara, H.; Okazaki, Y.; Kato, T. Proteomic analysis of lymphoblastoid cells derived from monozygotic twins discordant for bipolar disorder: A preliminary study. *PLoS ONE* **2013**, *8*, e53855. https://doi.org/10.1371/journal.pone.0053855.
64. Gedik, H.; Nguyen, T.H.; Peterson, R.E.; Chatzinakos, C.; Vladimirov, V.I.; Riley, B.P.; Bacanu, S.A. Identifying potential risk genes and pathways for neuropsychiatric and substance use disorders using intermediate molecular mediator information. *Front. Genet.* **2023**, *14*, 1191264. https://doi.org/10.3389/fgene.2023.1191264.
65. Merikangas, A.K.; Shelly, M.; Knighton, A.; Kotler, N.; Tanenbaum, N.; Almasy, L. What genes are differentially expressed in individuals with schizophrenia? A systematic review. *Mol. Psychiatry* **2022**, *27*, 1373–1383. https://doi.org/10.1038/s41380-021-01420-7.
66. Johansen, N.; Somasundaram, S.; Travaglini, K.J.; Yanny, A.M.; Shumyatcher, M.; Casper, T.; Cobbs, C.; Dee, N.; Ellenbogen, R.; Ferreira, M.; et al. Interindividual variation in human cortical cell type abundance and expression. *Science* **2023**, *382*, eadf2359. https://doi.org/10.1126/science.adf2359.
67. Kawabe, Y.; Seki, M.; Yoshimura, A.; Nishino, K.; Hayashi, T.; Takeuchi, T.; Iguchi, S.; Kusa, Y.; Ohtsuki, M.; Tsuyama, T.; et al. Analyses of the interaction of WRNIP1 with Werner syndrome protein (WRN) in vitro and in the cell. *DNA Repair* **2006**, *5*, 816–828. https://doi.org/10.1016/j.dnarep.2006.04.006.
68. Chen, D.T.; Jiang, X.; Akula, N.; Shugart, Y.Y.; Wendland, J.R.; Steele, C.J.M.; Kassem, L.; Park, J.H.; Chatterjee, N.; Jamain, S.; et al. Genome-wide association study meta-analysis of European and Asian-ancestry samples identifies three novel loci associated with bipolar disorder. *Mol. Psychiatry* **2013**, *18*, 195–205. https://doi.org/10.1038/mp.2011.157.

69. Cheung, M.; Abu-Elmagd, M.; Clevers, H.; Scotting, P.J. Roles of Sox4 in central nervous system development. *Brain Res. Mol. Brain Res.* **2000**, *79*, 180–191. https://doi.org/10.1016/s0169-328x(00)00109-1.
70. Mu, L.; Berti, L.; Masserdotti, G.; Covic, M.; Michaelidis, T.M.; Doberauer, K.; Merz, K.; Rehfeld, F.; Haslinger, A.; Wegner, M.; et al. SoxC transcription factors are required for neuronal differentiation in adult hippocampal neurogenesis. *J. Neurosci. Off. J. Soc. Neurosci.* **2012**, *32*, 3067–3080. https://doi.org/10.1523/JNEUROSCI.4679-11.2012.
71. Bergsland, M.; Werme, M.; Malewicz, M.; Perlmann, T.; Muhr, J. The establishment of neuronal properties is controlled by Sox4 and Sox11. *Genes. Dev.* **2006**, *20*, 3475–3486. https://doi.org/10.1101/gad.403406.
